# A λ-Carrageenan-Enriched Sulfated Galactan from *Gigartina radula* Attenuates Atopic Dermatitis via Coordinated Anti-Inflammatory and Immunomodulatory Mechanisms

**DOI:** 10.3390/md24030119

**Published:** 2026-03-22

**Authors:** Kexin Du, Shuo Liang, Zijing Wu, Yujing Wang, Pengcheng Gao, Wei Han, Youjing Lv, Guangli Yu, Guoyun Li

**Affiliations:** 1 Key Laboratory of Marine Drugs, Shandong Key Laboratory of Glycoscience and Glycotherapeutics, Ministry of Education, School of Medicine and Pharmacy, Ocean University of China, Qingdao 266003, China; 15206566923@163.com (K.D.); liangshuo980420@163.com (S.L.); wzj20000928@163.com (Z.W.); 15186970448@163.com (Y.W.); gaopengchengsir@163.com (P.G.); glyu@ouc.edu.cn (G.Y.); 2 Qingdao Key Laboratory of Respiratory Comorbidity Remodeling and Precision Prevention, Qingdao Municipal Hospital, Qingdao 260071, China; hanw@qdu.edu.cn; 3 Laboratory for Marine Drugs and Bioproducts, Qingdao Marine Science and Technology Center, Qingdao 266237, China

**Keywords:** atopic dermatitis, red alga, λ-carrageenan, sulfated galactan, anti-inflammatory, immunomodulation, marine polysaccharide

## Abstract

Atopic dermatitis (AD) is a chronic, relapsing inflammatory skin disease driven by immune dysregulation and epidermal barrier dysfunction. Current therapeutic options are often limited by safety concerns or suboptimal tolerability. In this study, we isolated and structurally characterized GRB-H—a λ-carrageenan-enriched sulfated hybrid galactan from the marine red alga *Gigartina radula*—as a complex polysaccharide containing κ-, ι-, μ-, ν-, and λ-carrageenan structural units, and systematically evaluated its anti-AD potential using both in vitro and in vivo models. In vitro, GRB-H significantly suppressed lipopolysaccharide (LPS)-induced nitric oxide (NO), tumor necrosis factor-α (TNF-α), and interleukin-6 (IL-6) in RAW 264.7 macrophages, and reduced 2,4-dinitrochlorobenzene (DNCB)-evoked TNF-α and IL-1β expression in HaCaT keratinocytes. In a DNCB-induced murine model of AD, topical application of GRB-H markedly ameliorated skin inflammation, epidermal hyperplasia, and dermal immune cell infiltration. GRB-H treatment lowered total serum immunoglobulin E (IgE) levels, restored the imbalanced Th1/Th2 cell ratio in the spleen, and downregulated the mRNA expression of key inflammatory cytokines—including TNF-α, IL-4, IL-5, IL-31, and interferon-γ (IFN-γ)—in lesional skin. Collectively, these findings demonstrate that GRB-H alleviates AD symptoms through coordinated local anti-inflammatory and systemic immunomodulatory actions, highlighting its promise as a marine-derived candidate for the topical management of AD.

## 1. Introduction

Atopic dermatitis (AD) is a chronic, relapsing inflammatory skin disorder clinically characterized by eczematous lesions, intense pruritus, and scaling [1]. Its rising global prevalence imposes a substantial burden on both patient quality of life and healthcare systems worldwide [2]. The pathogenesis of AD is complex and multifactorial, involving intricate interactions among skin barrier dysfunction, microbial dysbiosis, immune dysregulation, and genetic and environmental factors [3,4,5]. A predominant T-helper 2 (Th2) immune response is central to its immunopathology, driving the overproduction of cytokines such as interleukin-4 (IL-4), IL-13, and IL-31. These cytokines downregulate the expression of key barrier proteins like filaggrin [6], promote B cell class-switching to produce high levels of immunoglobulin E (IgE), and recruit inflammatory cells, including eosinophils and mast cells, into the skin, thereby perpetuating the disease cycle [7].

Current first-line clinical management of AD primarily relies on topical corticosteroids and calcineurin inhibitors [8]. However, long-term corticosteroid use is associated with local adverse effects such as skin atrophy and telangiectasia, as well as potential systemic risks. Calcineurin inhibitors, while effective, often cause transient burning and stinging, which can limit patient adherence [9]. For moderate-to-severe cases, systemic immunomodulators (e.g., cyclosporine) or biologic agents (e.g., dupilumab) represent advanced therapeutic options [10]. Nonetheless, concerns regarding side effects (e.g., hepatorenal toxicity) and high costs persist [9]. Therefore, the discovery of novel, naturally derived therapeutics with multi-target activities and favorable safety profiles remains a crucial objective in AD research.

Natural polysaccharides have attracted increasing attention for their multifaceted potential in managing AD. Their bioactivities span epidermal barrier enhancement, immunomodulation, microbiome regulation, moisturization, and anti-inflammatory effects [11]. Dysfunctional keratinocytes are central to the disrupted skin barrier in AD, and natural polysaccharides can protect and restore their function through diverse mechanisms. For example, a polysaccharide from *Sargassum fusiforme* suppresses the expression of matrix metalloproteinases and inflammatory cytokines (IL-6, IL-1β, TNF-α), alleviating UVA-induced inflammation and oxidative damage in keratinocytes [12]. A high β-D-glucan polysaccharide from *Typha latifolia* L. fruit promotes keratinocyte differentiation and supports normal barrier formation by upregulating Smad3 and PKC-α expression [13]. Fucoidan SHC4-6 from *Sargassum horneri* enhances tight-junction protein expression in HaCaT cells, protecting them from injury by particulate matter [14]. Sacran, a polysaccharide from *Aphanothece sacrum*, upregulates profilaggrin expression in an AD-like mouse model. Profilaggrin is hydrolyzed into functional filaggrin, which directly contributes to barrier repair and inflammation mitigation [15]. Furthermore, Sacran can directly bind and neutralize IL-1α released by keratinocytes, blocking the inflammatory cascade initiated by this alarmin after barrier disruption, thereby reducing cellular damage and oxidative stress [16]. The immunopathological core of AD is a skewed Th2-dominant response. Research indicates that topical application of algal polysaccharides derived from *C. okamuranus* promotes tolerogenic dendritic-cell differentiation, which subsequently activates regulatory T cells. This process downregulates Th2-mediated immune responses and related cytokines (e.g., TSLP, IL-5, IL-33), while also reducing mast-cell degranulation [17].

As an important class of natural polysaccharides, algal polysaccharides have been extensively reported to exhibit diverse bioactivities, including anti-wrinkle, whitening, moisturizing, UV-protective, antioxidant, and anti-inflammatory properties [18]. These multifunctional characteristics underscore their promising potential in the therapy of AD [17,18,19,20]. Studies on sulfated polysaccharides, in particular, highlight their potent immunomodulatory and anti-inflammatory effects, which are often structure-dependent. For instance, sulfated galactans from *Chondrus verrucosus* with higher sulfate content (25.3% and 28.1%) demonstrated stronger hyaluronidase inhibition and more effectively suppressed calcium ionophore A23187-induced mast-cell degranulation, suggesting a positive correlation between sulfate content and anti-allergic inflammatory activity [21]. In animal models, a sulfated polysaccharide fraction (PLS) from *Gracilaria caudata* showed broad-spectrum anti-inflammatory and analgesic effects. Intraperitoneal injection of PLS dose-dependently inhibited carrageenan-, dextran-, and histamine-induced paw edema in mice, and significantly reduced total leukocyte count and neutrophil infiltration in a carrageenan-induced peritonitis model. It also decreased myeloperoxidase (MPO) activity, downregulated TNF-α and IL-1β levels in peritoneal fluid, and alleviated inflammatory hyperalgesia [22]. Similarly, sulfated polysaccharides from *Gracilaria intermedia* were shown to mitigate carrageenan-induced paw edema and effectively inhibit neutrophil migration by modulating IL-1β production [23]. At the molecular level, a sulfated polysaccharide (GNP) from *Gelidium crinale* suppressed the expression of inducible nitric oxide synthase (iNOS) and cyclooxygenase-2 (COX-2), and reduced pro-inflammatory cytokine production in LPS-stimulated RAW 264.7 macrophages by blocking the MAPK/NF-κB signaling pathway [24].

In this context, the present study reports, for the first time, the therapeutic potential of a highly sulfated galactan, GRB-H, extracted from the marine red alga *Gigartina radula*, against AD. Through complementary in vivo experiments, we demonstrate that GRB-H not only significantly alleviates DNCB-induced AD-like skin symptoms in mice and reduces total serum IgE levels, but also restores the Th1/Th2 immune balance in the spleen. Furthermore, in vitro assays revealed that GRB-H directly suppressed the release of pro-inflammatory cytokines (TNF-α, IL-6, and IL-1β) in both LPS-stimulated macrophages and DNCB-stimulated keratinocytes. These findings provide novel experimental evidence supporting the development of GRB-H as a promising marine-derived candidate for the topical treatment of AD.

## 2. Results

### 2.1. Physicochemical Characterization of GRB-H

**Figure 1:**
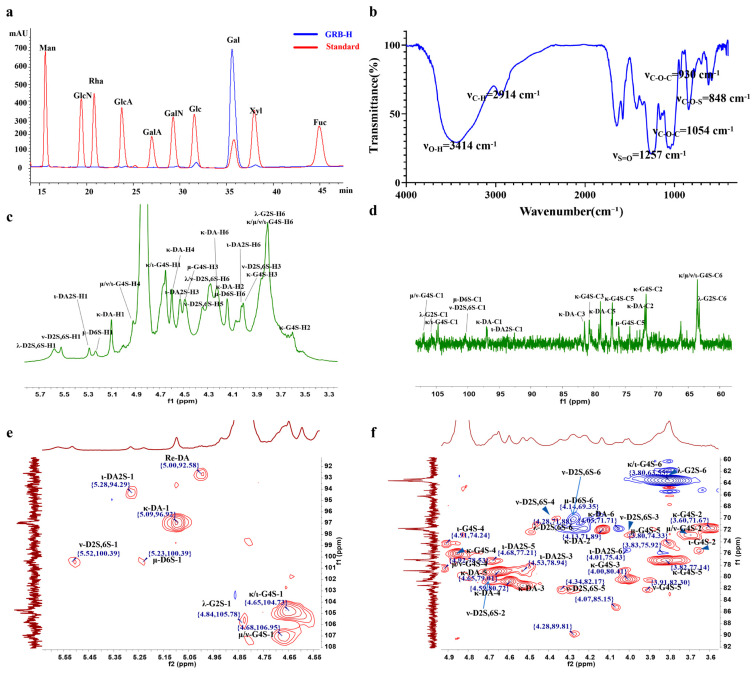
Preliminary structural analysis of GRB-H. (**a**) Monosaccharide composition. (**b**) FTIR spectrum. (**c**,**d**) ^1^H and ^13^C NMR spectra. (**e**,**f**) HSQC NMR spectra.

The extraction yield of GRB-H was 37.98% (*w*/*w*). Chemical composition analysis revealed that GRB-H consisted of 53.83% total sugars, 4.57% protein, and 34.35% sulfate. Monosaccharide composition analysis (Figure 1a) indicated that GRB-H was primarily composed of galactose (95.74%) with a minor amount of glucose (4.26%). The 3,6-anhydro-galactose content was 24.41% of the total sugar content. Due to the high viscosity of GRB-H, which precludes direct molecular weight determination by conventional gel permeation chromatography (GPC), the apparent viscosity was measured using a rotational viscometer. At a concentration of 15 mg/mL and 60 °C, the viscosity of GRB-H was determined to be 182.9 mPa·s. These results confirm that GRB-H is a highly sulfated galactan with a high molecular weight.

### 2.2. Fourier Transform Infrared (FTIR) Spectral Analysis of GRB-H

The FTIR spectrum of GRB-H displayed characteristic absorption bands (Figure 1b): a broad band at 3414 cm^−1^ was attributed to O–H stretching vibrations of the sugar rings; the band at 2941 cm^−1^ arose from C–H stretching of the methylene group at the C6 position; and the strong and broad absorption bands in the region of 1200–1000 cm^−1^ are attributed to the C–O–C and C–O–H stretching vibrations, along with the skeletal vibrations of the sugar rings. These three sets of signals are typical of polysaccharide sugar rings. Additional bands were observed at 1257 cm^−1^ (assigned to S=O stretching), 930 cm^−1^ (assigned to C–O–C stretching of the 3,6-anhydro-galactose ring), and 848 cm^−1^ (assigned to C–O–S stretching). The band at 848 cm^−1^ indicates the presence of a β-linked galactose residue sulfated at the C4 position, which is characteristic of κ/ι-type carrageenan.

### 2.3. Structural Composition Analysis of GRB-H

Based on the NMR spectra, the structural composition of GRB-H was further analyzed. Using 4,4-dimethyl-4-silapentane-1-sulfonic acid (DSS), recommended by the International Union of Pure and Applied Chemistry (IUPAC) as an internal standard, its main peak was set at a chemical shift of 0.00 ppm for reference.

In the ^1^H NMR spectrum (Figure 1c), proton signals from the sugar rings were primarily distributed within the 3.00–6.00 ppm range. Specifically, the H2–H6 proton peaks of the sugar rings were mostly concentrated between 3.00 and 4.50 ppm, showing significant signal overlap. Characteristic peak analysis revealed the absence of the distinct signal for α-L-3,6-anhydrogalactose (α-L-DA-H1) at 5.14 ppm, indicating that GRB-H does not contain agarose-like structures. In contrast, clear characteristic peaks were observed at 5.09 ppm (κ-DA-H1), 5.23 ppm (μ-D6S-H1), 5.28 ppm (ι-DA2S-H1), 5.52 ppm (ν-D2S,6S-H1), and 5.58 ppm (λ-D2S,6S-H1), demonstrating that GRB-H is a carrageenan-type galactan composed of the five structural units mentioned above.

In the ^13^C NMR spectrum (Figure 1d), carbon signals from the sugar rings appeared in the 60–110 ppm range, with the anomeric carbon signals located between 90 and 110 ppm, containing crucial structural information about the sugar chains. The HSQC NMR spectra (Figure 1e,f) further revealed characteristic signals corresponding to κ-, ι-, μ-, ν-, and λ-carrageenan. Detailed signal assignments are summarized in Table 1 [25,26].

Using the integrated peak area of the DSS internal standard as 1, the signal integrals of the five carrageenan units were normalized to calculate their relative contents. The results showed that the relative contents of κ-, ι-, μ-, ν-, and λ-carrageenan in GRB-H were 36.82%, 12.53%, 7.18%, 13.05%, and 30.42%, respectively, indicating that GRB-H is a hybrid galactan rich in λ-carrageenan. A schematic diagram of the carrageenan structural units of GRB-H is provided in Figure S1.

### 2.4. GRB-H Inhibits Inflammation in Macrophages and Keratinocytes

**Figure 2:**
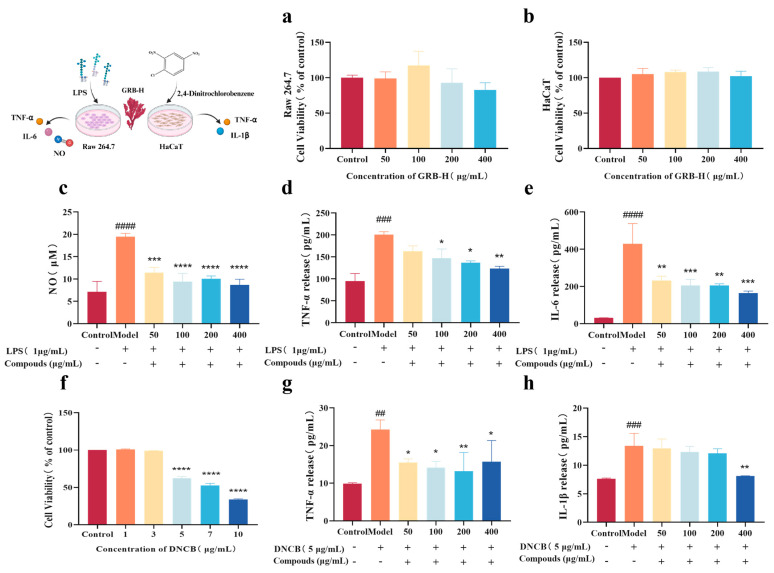
GRB-H inhibits cellular inflammation. (**a**,**b**) Cytotoxicity of GRB-H on RAW 264.7 and HaCaT cells. (**c**) Effect of GRB-H on NO production in LPS-induced RAW 264.7 cells. (**d**,**e**) Effect of GRB-H on TNF-α and IL-6 release in LPS-induced RAW 264.7 cells. (**f**) Effect of DNCB on HaCaT cell viability. (**g**,**h**) Effect of GRB-H on TNF-α and IL-1β release in DNCB-induced HaCaT cells. Data are presented as mean ± SEM (*n* = 3). –/+, absence/presence. The upper row indicates LPS or DNCB treatment, and the lower row indicates compounds treatment. * *p* < 0.05, ** *p* < 0.01, *** *p* < 0.001, **** *p* < 0.0001 (Treatment vs. Model group). ^##^ *p* < 0.01, ^###^ *p* < 0.001, ^####^ *p* < 0.0001 (Model vs. Control group).

The cytotoxicity of GRB-H was first evaluated using the CCK-8 assay. The results showed that GRB-H exhibited no significant cytotoxicity toward RAW 264.7 macrophages or HaCaT cells at concentrations up to 400 µg/mL (Figure 2a,b). 2,4-Dinitrochlorobenzene (DNCB), a hapten commonly used to induce an atopic dermatitis-like inflammatory response, can trigger inflammation in keratinocytes. A concentration of 5 µg/mL DNCB was selected for subsequent experiments, as it did not significantly affect cell viability at or below 3 µg/mL (Figure 2f).

We next investigated the anti-inflammatory effects of GRB-H. In LPS-stimulated RAW 264.7 macrophages, GRB-H significantly inhibited NO production at concentrations as low as 50 µg/mL (Figure 2c). The IC_50_ value of GRB-H was determined to be 136.2 μg/mL, providing a reasonable reference for its anti-inflammatory activity. Furthermore, ELISA analysis demonstrated that GRB-H dose-dependently suppressed the LPS-induced release of the pro-inflammatory cytokines TNF-α and IL-6 (Figure 2d,e). Similarly, in DNCB-stimulated HaCaT keratinocytes, GRB-H (50 µg/mL) significantly inhibited the release of TNF-α, while a significant reduction in IL-1β release was achieved at the higher concentration of 400 µg/mL (Figure 2g,h).

### 2.5. GRB-H Alleviates DNCB-Induced AD Symptoms in Mice

**Figure 3:**
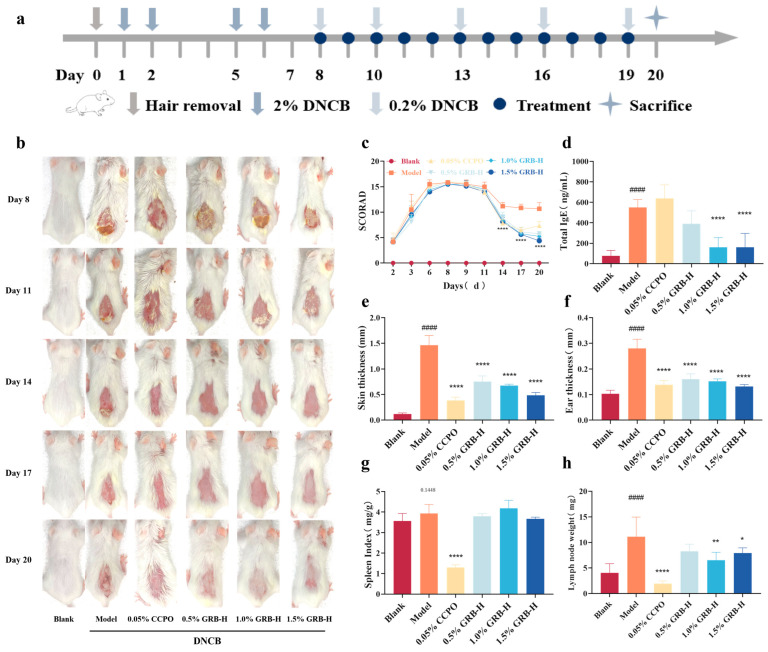
Effects of GRB-H on DNCB-induced atopic dermatitis symptoms in mice. (**a**) Experimental timeline. (**b**) Representative photographs of dorsal skin. (**c**) Dermatitis severity score over time. (**d**) Serum total IgE levels. (**e**) Dorsal skin thickness on day 20. (**f**) Right ear thickness on day 20. (**g**) Spleen index. (**h**) Bilateral inguinal lymph node weight. Data are presented as mean ± SEM (*n* = 6). * *p* < 0.05, ** *p* < 0.01, **** *p* < 0.0001 (Treatment vs. Model group). ^####^ *p* < 0.0001 (Model vs. Blank group).

The experimental timeline is illustrated in Figure 3a. Topical DNCB sensitization successfully induced AD-like symptoms, including increased ear thickness, skin erythema, edema, and crusting (Figure 3b). These symptoms were accompanied by a significant increase in the clinical dermatitis score and lesional skin thickness compared to the Blank group (Figure 3c,e,f). Treatment with GRB-H (0.5%, 1.0%, and 1.5%) or the positive control CCPO (0.05% clobetasol propionate ointment) significantly ameliorated these macroscopic symptoms and reduced ear thickness (Figure 3b,c,e,f).

Serum total IgE level, a key biomarker of allergic sensitization and AD severity, was markedly elevated in the model group. GRB-H treatment dose-dependently reduced serum IgE levels (Figure 3d). Notably, CCPO treatment did not significantly alter the elevated IgE level. The model group also exhibited a trend of increased spleen index and pronounced enlargement of inguinal lymph nodes, indicative of systemic immune activation [27]. GRB-H treatment, particularly at the 1.0% and 1.5% doses, significantly attenuated lymph node enlargement (Figure 3h) and showed a trend toward reducing the spleen index (Figure 3g). In contrast, CCPO treatment caused significant atrophy of both the spleen and lymph nodes, likely reflecting the broad side effects of this potent glucocorticoid [28].

### 2.6. Histopathological Analysis of Skin Lesions in Mice

**Figure 4:**
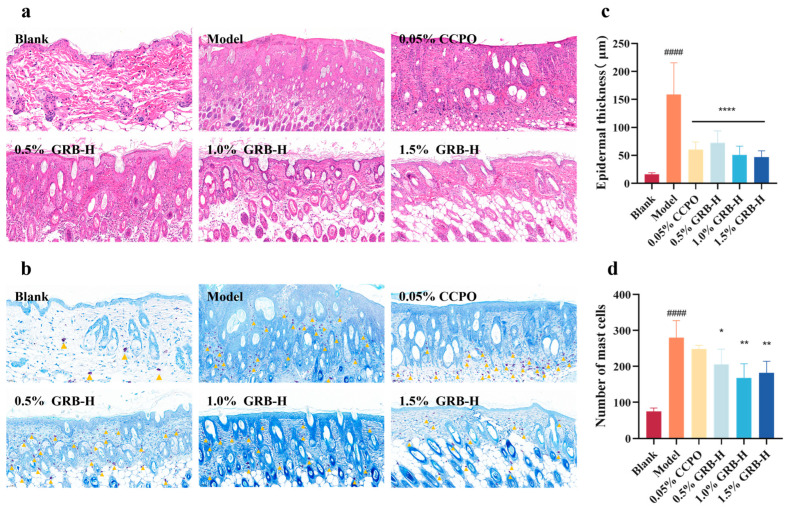
Histopathological evaluation of skin lesions. (**a**) Representative H&E-stained sections (40× for Blank group, 20× for others). (**b**) Representative TB-stained sections showing mast cells (indicated by yellow triangles; 40× for Blank group, 20× for others). (**c**) Quantification of epidermal thickness. (**d**) Quantification of mast cell numbers per field. Data are presented as mean ± SEM (*n* = 5 fields per mouse). * *p* < 0.05, ** *p* < 0.01, **** *p* < 0.0001 (Treatment vs. Model group). ^####^ *p* < 0.0001 (Model vs. Blank group).

Histopathological analysis of dorsal skin lesions revealed that DNCB-induced AD mice exhibited typical pathological features, including hyperkeratosis, significant epidermal hyperplasia (acanthosis), and dermal inflammatory cell infiltration (Figure 4a). Both CCPO and GRB-H treatments significantly ameliorated epidermal hyperplasia (Figure 4a,c). Toluidine blue staining showed a marked increase in mast cell infiltration in the dermis of the model group, which was significantly reduced by topical GRB-H application (Figure 4b,d).

### 2.7. GRB-H Suppresses Inflammatory Cytokine Expression in Skin Lesions

**Figure 5:**
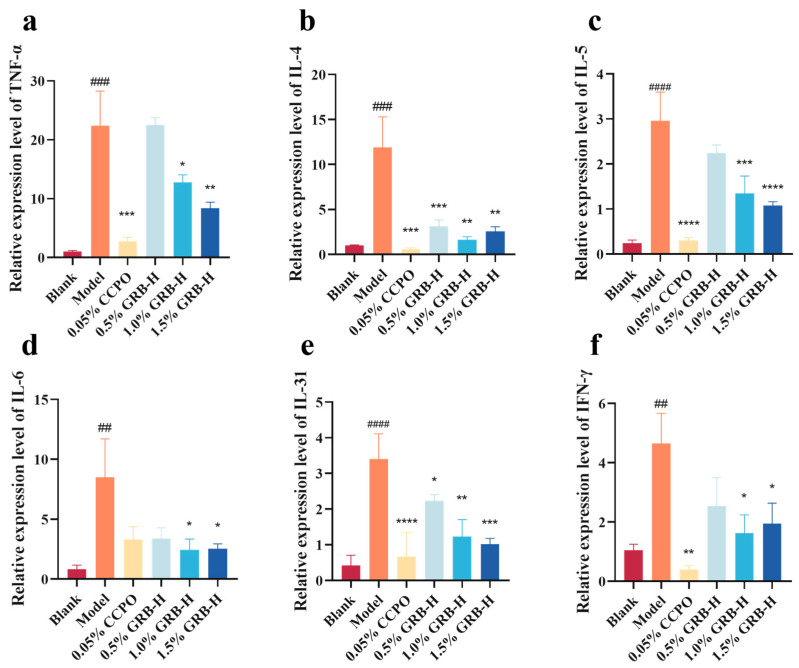
mRNA expression of inflammatory cytokines in skin lesions. Relative mRNA levels of (**a**) TNF-α, (**b**) IL-4, (**c**) IL-5, (**d**) IL-6, (**e**) IL-31, and (**f**) IFN-γ were determined by RT-qPCR and normalized to GAPDH. Data are presented as mean ± SEM (*n* = 3). * *p* < 0.05, ** *p* < 0.01, *** *p* < 0.001, **** *p* < 0.0001 (Treatment vs. Model group). ^##^ *p* < 0.01, ^###^ *p* < 0.001, ^####^ *p* < 0.0001 (Model vs. Blank group).

To further elucidate the local anti-inflammatory effect of GRB-H, we analyzed the mRNA expression of key inflammatory cytokines in the skin lesions by RT-qPCR. The expression levels of TNF-α, IL-4, IL-5, IL-6, IL-31, and IFN-γ were significantly upregulated in the model group compared to the Blank group. Topical treatment with GRB-H markedly suppressed the mRNA expression of all these cytokines (Figure 5).

### 2.8. GRB-H Modulates Systemic Th1/Th2 Immune Balance

**Figure 6:**
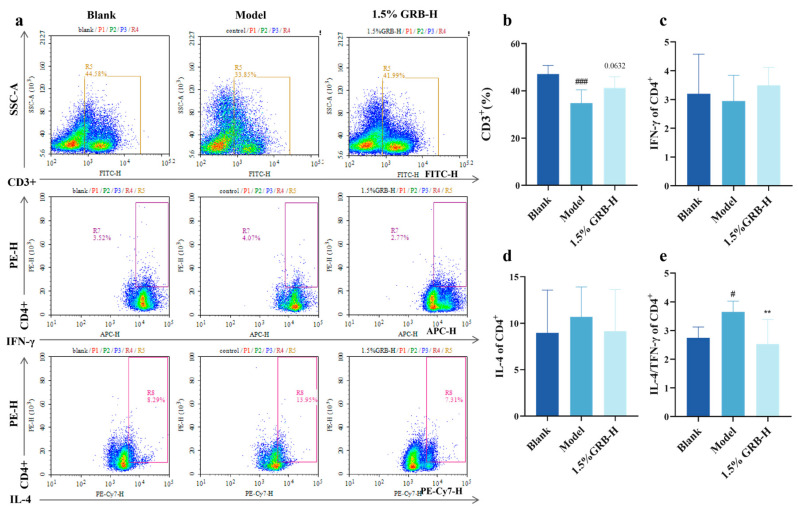
Effect of GRB-H on splenic T cell responses. (**a**) Sorting results of the single-cell suspension. (**b**) Proportion of CD3^+^ T cells. (**c**) Proportion of IFN-γ^+^ (Th1) cells within CD3^+^CD4^+^ T cells. (**d**) Proportion of IL-4^+^ (Th2) cells within CD3^+^CD4^+^ T cells. (**e**) Th2/Th1 ratio calculated from (**c**,**d**). Data are presented as mean ± SEM (*n* = 6). ** *p* < 0.01 (Treatment vs. Model group). ^#^ *p* < 0.05, ^###^ *p* < 0.001 (Model vs. Blank group).

Given the systemic immunomodulatory effects suggested by reduced serum IgE and lymphoid organ changes, we next investigated the effect of GRB-H on T-helper cell polarization in the spleen. Flow cytometric analysis was performed on splenocytes from the Blank, Model, and high-dose GRB-H (1.5%) groups. As shown in Figure 6b, compared with the Blank group, the proportion of CD3^+^ T cells was significantly decreased in the Model group. Although not statistically significant (*p* = 0.0632), treatment with 1.5% GRB-H showed a trend toward increasing the CD3^+^ T cell population.

Although the absolute percentages of Th1 (IFN-γ^+^) and Th2 (IL-4^+^) cells within CD4^+^ T cells showed trends but not statistically significant changes (Figure 6c,d), the Th2/Th1 ratio, a critical indicator of immune skewing, was significantly increased in the Model group and was markedly reduced by GRB-H treatment (Figure 6e). This indicates that GRB-H can correct the Th2-dominant immune imbalance associated with AD at the systemic level.

## 3. Discussion

This study provides the first systematic evidence that GRB-H, a highly sulfated galactan from the marine red alga *Gigartina radula*, exerts potent anti-inflammatory and immunomodulatory effects, significantly ameliorating AD in both cellular and mouse models. Our findings demonstrate that GRB-H alleviates local skin pathology and modulates key systemic immune disturbances in AD, supporting its potential as a novel topical therapeutic candidate.

A growing body of the literature demonstrates that carrageenans naturally occur as hybrid structures rather than idealized homogeneous polymers. A recent comprehensive study analyzing commercial λ-carrageenan samples found that none of the six samples contained pure λ-type structural elements; instead, they contained κ-, ι-, and ν-units [29]. This observation aligns with reports on various red algae, where hybrid carrageenans have been consistently identified. For instance, the polysaccharide extracted from *Gymnogongrus tenuis* was characterized as a hybrid predominantly composed of ι-carrageenan, with κ- and ν-type units also present [30]. Similarly, the cold-water extract from *Chondracanthus chamissoi* was found to comprise 35% κ-type, 43% ι-type, and 22% μ-type carrageenan [31]. In *Gigartina skottsbergii* and *Sarcothalia crispata*, the polysaccharides were primarily composed of κ-, ι-, μ-, and ν-carrageenan motifs [32]. These findings collectively demonstrate that carrageenans isolated from red algae frequently exhibit hybrid structural features, with multiple repeating units coexisting within the same polysaccharide preparation. The structural complexity of GRB-H, therefore, is consistent with this well-documented natural phenomenon.

The relatively high viscosity of GRB-H (182.9 mPa·s at 15 mg/mL and 60 °C) suggests a considerable molecular size, consistent with the typical characteristics of carrageenan-type polysaccharides. Although direct molecular weight determination by GPC was not feasible due to viscosity-related technical limitations, an estimation can be made based on data from the literature. λ-Carrageenan has been reported to have a weight-average molecular weight (Mw) ranging from 340 to 870 kDa [33], while κ/ι-hybrid carrageenans exhibit Mw values between 1020 and 2250 kDa [34]. Considering the λ-enriched nature of GRB-H and its viscosity behavior, the molecular weight of GRB-H is estimated to be approximately 600–800 kDa. This estimated range is within the expected values for sulfated galactans and supports the polymeric nature of GRB-H.

The bioactivity of GRB-H is likely underpinned by its unique chemical structure. With a high sulfate content (~34%) and a substantial proportion of 3,6-anhydro-galactose (~24%), GRB-H exhibits structural features strongly associated with anti-inflammatory and immunomodulatory properties in polysaccharides [35,36,37]. Sulfate groups are known to mediate interactions with pattern recognition receptors (e.g., Toll-like receptor 4) and negatively regulate downstream pro-inflammatory signaling pathways such as MAPK and NF-κB [38,39]. The 3,6-anhydro-galactose moiety is a characteristic structure of red algal galactans that often contributes to their specific conformation and bioactivity [40,41]. Consistently, we found that GRB-H effectively inhibited LPS-induced production of NO, TNF-α, and IL-6 in RAW 264.7 macrophages, as well as DNCB-induced generation of TNF-α and IL-1β in HaCaT keratinocytes, suggesting its direct interference with classical inflammatory pathways.

Furthermore, oxidative stress is a central pathogenic factor in atopic dermatitis (AD), forming a self-amplifying vicious cycle with skin barrier dysfunction and immune inflammation [42,43]. Elevated levels of reactive oxygen species (ROS) can directly damage keratinocytes, disrupt epidermal integrity, and induce cellular autophagy and apoptosis through pathways such as MAPK activation and mTOR inhibition, ultimately leading to epidermal homeostasis imbalance [42,43]. Meanwhile, the Th2-dominant inflammatory milieu in AD (characterized by elevated IL-4 and IL-13) promotes ROS generation; conversely, ROS themselves upregulate pro-inflammatory cytokine expression and enhance lipid peroxidation, thereby perpetuating disease progression [44]. Therefore, targeting oxidative stress represents a promising therapeutic strategy for AD. In this context, the anti-inflammatory activity of GRB-H observed in our study may be indirectly linked to its potential to modulate oxidative stress-related pathways, although direct antioxidant effects were not examined. Galactans derived from red algae have been widely reported to exhibit antioxidant activities that are closely dependent on their fine structural features [45,46]. Future studies are warranted to investigate whether GRB-H possesses similar antioxidant properties and whether such activities contribute to its anti-inflammatory effects in AD.

In the DNCB-induced AD mouse model, topical GRB-H application demonstrated multidimensional therapeutic efficacy. It significantly improved core AD phenotypes, including clinical severity scores, skin/ear thickening, epidermal hyperplasia, and inflammatory cell infiltration (Figure 3 and Figure 4). Crucially, GRB-H’s effects extended beyond local anti-inflammation. AD is characterized by systemic Th2 skewing and allergic sensitization [47]. We found that GRB-H significantly and dose-dependently reduced the elevated serum total IgE levels in AD mice (Figure 3d), a hallmark of its systemic immunomodulatory action. Furthermore, the attenuation of lymph node enlargement and the trend toward a normalized spleen index by GRB-H (Figure 3g,h) suggest a mitigation of systemic immune activation and lymphoid hyperplasia. Importantly, unlike the potent glucocorticoid CCPO, which induced immune organ atrophy—a sign of broad immunosuppression—GRB-H achieved therapeutic benefits without such apparent systemic toxicity, highlighting a potentially superior safety profile.

At the molecular level, GRB-H likely exerts its effects by modulating the dysregulated cytokine network in AD. The pathogenesis of AD involves a complex interplay of cytokines. A shift toward Th2 cytokines (e.g., IL-4, IL-5, IL-13, IL-31) drives IgE production, eosinophil recruitment, and barrier dysfunction [7,48,49]. Additionally, IL-31 can further amplify the inflammatory cascade by delaying eosinophil apoptosis and stimulating them to secrete pro-inflammatory mediators such as IL-1β, IL-6, CXCL1, CXCL8, CCL2, and CCL18 [50]. Meanwhile, Th1 cytokines like IFN-γ and the pro-inflammatory mediator TNF-α contribute to chronic skin inflammation and immune cell infiltration [51]. Our data show that GRB-H significantly suppressed the mRNA expression of TNF-α, IL-4, IL-5, IL-6, IL-31, and IFN-γ in lesional skin (Figure 5), correlating with the observed local anti-inflammatory and histopathological improvements. Moreover, flow cytometry analysis revealed that GRB-H treatment significantly reduced the elevated Th2/Th1 ratio in the spleen (Figure 6e), indicating a correction of the systemic immune imbalance. Therefore, we propose that GRB-H functions through a dual mechanism: locally, it inhibits the production of a broad spectrum of inflammatory mediators in the skin; systemically, it modulates T-cell differentiation to restrain excessive Th2 responses and consequent IgE production. This multi-target, immunomodulatory approach may offer a more targeted and potentially safer alternative to the broad immunosuppression caused by conventional corticosteroids.

Several limitations of this study should be acknowledged. First, the precise molecular targets and intracellular signaling pathways (e.g., TLR4/MyD88/NF-κB, JAK-STAT) through which GRB-H acts require further investigation using techniques like receptor blockade, siRNA knockdown, or Western blotting. Second, besides Th1/Th2 cells, other immune players such as Th17 and Treg cells are also crucial in AD pathogenesis [52,53,54]. The effects of GRB-H on these cell types remain to be explored. Third, this study employed topical administration; the skin permeability, metabolic fate, and bioavailability of GRB-H are currently unknown. Future studies focusing on formulation development and pharmacokinetics will be essential for its clinical translation. Fourth, although hybrid carrageenans are widely present in nature, the exact covalent connectivity among the different structural units within GRB-H—whether they constitute a true hybrid molecule or a mixture of distinct carrageenans—requires further validation using advanced techniques such as fractionation studies.

## 4. Materials and Methods

### 4.1. Materials and Reagents

*Gigartina radula* was supplied by Qingdao Jüdàyáng Algae Industry Group (Qingdao, China), sourced from Chile. The Nitric Oxide Assay Kit was purchased from Beyotime (Shanghai, China). The Omni-easy™ Ready-to-Use BCA Protein Assay Kit was obtained from Yeasen Biotechnology Co., Ltd (Shanghai, China). The Mouse IgE ELISA Kit was procured from FineTest (Wuhan, China). The Cell Counting Kit-8 (CCK-8) was acquired from LANCOSA (Jinan, China). The ABScript III RT Mix for qPCR with gDNA remover and 2× Universal SYBR Green qPCR Mix were from ABclonal Technology (Wuhan, China). The murine macrophage cell line RAW 264.7 and the human keratinocyte cell line HaCaT were acquired from the China Center for Type Culture Collection (CCTCC, Wuhan, China). All other chemicals were of analytical grade or higher and were purchased from commercial suppliers.

### 4.2. Extraction and Physicochemical Characterization of GRB-H Polysaccharide

Cleaned, dried, and powdered *Gigartina radula* was mixed with distilled water at a solid-to-liquid ratio of 1:30 (*w*/*v*) and extracted in a 80 °C water bath for 2 h. After centrifugation, the supernatant was collected. The residue was re-extracted once under the same conditions. The combined supernatants were concentrated under reduced pressure, and polysaccharides were precipitated by adding four volumes of 95% (*v*/*v*) ethanol, followed by overnight storage at 4 °C. The precipitate was collected by centrifugation, redissolved in distilled water, dialyzed (molecular weight cutoff: 3.5 kDa) against distilled water for 48 h, and finally freeze-dried to obtain the crude GRB-H polysaccharide.

The total sugar content was determined by the phenol–sulfuric acid method using D-galactose as the standard. Protein content was measured using the BCA assay. Sulfate content was analyzed by the gelatin–barium chloride turbidimetric method. The 3,6-anhydro-galactose content was quantified via the resorcinol method. Monosaccharide composition analysis was performed according to a previously reported method with slight modifications [55]. Briefly, GRB-H samples were hydrolyzed with 4 M trifluoroacetic acid (TFA) at 110 °C for 4 h. After removing excess TFA by co-evaporation with methanol, the hydrolyzates were derivatized with 0.5 M 1-phenyl-3-methyl-5-pyrazolone (PMP). The PMP derivatives were separated on an Agilent 1100 HPLC system equipped with a Hypersil BDS C18 column (250 mm × 4.6 mm, 5 μm). The mobile phase consisted of 0.1 M phosphate buffer (pH 6.7) and acetonitrile (83:17, *v*/*v*) at a flow rate of 0.8 mL/min, with detection at 245 nm.

Viscosity of GRB-H (15 mg/mL in deionized water) was measured at 60 °C using an NDJ-5S rotational viscometer (Shanghai Precision Instrument Co., Ltd., Shanghai, China). Measurements were performed in triplicate, and results expressed as mean (mPa·s).

### 4.3. Fourier Transform Infrared (FTIR) Spectroscopy of GRB-H

GRB-H was thoroughly ground and homogenized with potassium bromide (KBr) at an appropriate ratio, then pressed into a transparent pellet using a mechanical press. The FTIR spectrum was recorded on a Nicolet iS50 spectrometer (Thermo Fisher Scientific, Waltham, MA, USA) over a scanning range of 4000–400 cm^−1^.

### 4.4. Nuclear Magnetic Resonance (NMR) Analysis of GRB-H

To elucidate the structural characteristics of GRB-H, nuclear magnetic resonance (NMR) analysis was performed. The sample preparation was conducted as follows: approximately 70 mg of GRB-H was dissolved in distilled water to prepare a 10 mg/mL solution. The solution was then partially depolymerized by adding sulfuric acid (H_2_SO_4_) to a final concentration of 0.1 M and incubating at 60 °C for 0.5 h. Following neutralization, the reaction mixture was subjected to ultrafiltration using a membrane with a molecular weight cutoff (MWCO) of 30 kDa to remove low-molecular-weight salts and acid-hydrolyzed fragments. The retentate was collected and lyophilized for subsequent NMR analysis.

NMR spectra were acquired on a 600 MHz spectrometer at 25 °C. Deuterium oxide (D_2_O) was used as the solvent, and 4,4-dimethyl-4-silapentane-1-sulfonic acid (DSS) served as the internal reference. High-resolution ^1^H NMR, ^13^C NMR and HSQC spectra of GRB-H were recorded.

### 4.5. Cell Viability and Anti-Inflammatory Activity

RAW 264.7 and HaCaT cells were cultured under standard conditions (high-glucose DMEM with 10% FBS, 37 °C, 5% CO_2_). After seeding in 96-well plates (5 × 10^4^ cells/well) for 24 h, cells were treated with serial concentrations of GRB-H (0–400 μg/mL). For the viability assessment in HaCaT cells, the treatment medium also contained a gradient of DNCB (1, 3, 5, 7, 10 μg/mL). For anti-inflammatory evaluation, RAW 264.7 cells were co-stimulated with 1 μg/mL LPS, while HaCaT cells were co-stimulated with 5 μg/mL DNCB; both sets were treated alongside GRB-H for 24 h. The culture supernatant was collected to assess inflammatory markers: NO production was analyzed via the Griess method, and the IC_50_ value of GRB-H for NO inhibition in LPS-stimulated RAW 264.7 cells was calculated by nonlinear regression analysis using GraphPad Prism 9.0. The levels of TNF-α, IL-6, and IL-1β were determined using corresponding ELISA kits (Invitrogen, Carlsbad, CA, USA).

### 4.6. Establishment of AD Mouse Model and Observation of Macroscopic Indicators

Thirty-six female BALB/c mice (5 weeks old, weight 20 ± 2 g) were purchased from GemPharmatech Co., Ltd. (Nanjing, China) and housed in an SPF-grade animal facility under controlled temperature (22 ± 2 °C) and relative humidity (50–70%). All animal experiments were approved by the Experimental Animal Ethics Committee of the Ocean University of China (License No.: SCXK-2023-0009; Approval No.: OUC-SMP-2025-08-04).

The mice were randomly divided into six groups (*n* = 6 per group): a normal control group (Blank), an atopic dermatitis model group (Model), a positive control group treated with 0.05% clobetasol propionate ointment (0.05% CCPO), and three GRB-H treatment groups receiving low (0.5% GRB-H), medium (1.0% GRB-H), and high (1.5% GRB-H) doses.

The AD mouse model was established as follows: after shaving the dorsal hair (approximately 2 cm × 2 cm), mice were sensitized by topical application of a 2% DNCB solution (in acetone:olive oil = 3:1) on the shaved back and the right ear on day 1. From day 8 to day 19, the treatment groups received daily topical application of 200 μL of the respective GRB-H solution or 0.05% CCPO ointment onto the lesional skin, while the Blank and Model groups received an equal volume of normal saline (Figure 3a).

The dermatitis severity of the dorsal skin was assessed on days 2, 3, 6, 8, 9, 11, 14, 17, and 20 using a clinical scoring system based on four parameters: erythema, edema, excoriation, and scaling/dryness. Each parameter was graded on a scale of 0 (none) to 4 (severe), and the individual scores were summed to obtain the total dermatitis score per mouse. On day 20, all mice were euthanized. The lesional dorsal skin, right ear, spleen, inguinal lymph nodes, and blood were collected. Skin thickness at the center of the dorsal lesion and the middle of the right ear was measured using a digital caliper. The spleen and bilateral inguinal lymph nodes were weighed, and the spleen index was calculated as spleen weight (mg) divided by body weight (g).

### 4.7. Histopathological Examination

The collected dorsal lesional skin tissues were fixed in 4% paraformaldehyde, embedded in paraffin, and sectioned at a thickness of 6 μm. The sections were stained with Hematoxylin and Eosin (H&E) or Toluidine Blue (TB) according to standard protocols. Stained sections were observed and imaged under a light microscope (Olympus, Tokyo, Japan). Epidermal thickness and the number of mast cells in the dermis were quantified in five randomly selected fields per section using ImageJ software 1.53m (National Institutes of Health, Bethesda, MD, USA).

### 4.8. ELISA for Serum IgE

The total immunoglobulin E (IgE) concentration in the serum samples was determined using a commercial Mouse IgE ELISA Kit (FineTest, Wuhan, China) according to the manufacturer’s instructions.

### 4.9. RT-qPCR of Dorsal Lesional Skin

Approximately 50 mg of dorsal lesional skin tissue was homogenized in Trizol reagent to extract total RNA. One microgram of total RNA was reverse-transcribed into cDNA using the ABScript III RT Mix kit (ABclonal Technology, Wuhan, China). Quantitative real-time PCR (qPCR) was performed on a QuantStudio™ Desian & Analysis Software v1.5.2 using 2× Universal SYBR Green qPCR Mix (ABclonal Technology, Wuhan, China). GAPDH was used as the internal reference gene, and the relative mRNA expression levels of target genes were calculated using the 2^−ΔΔCt^ method. The primer sequences used are listed in Table S1.

### 4.10. Splenocyte Isolation and Flow Cytometry

Spleens were aseptically removed and gently ground through a 70 μm cell strainer to obtain a single-cell suspension. Red blood cells were lysed using ACK lysis buffer. Cell viability was assessed, and dead cells were excluded by staining with Fixable Viability Dye 545. For surface staining, cells were incubated with fluorescently labeled antibodies against mouse CD45 (PerCP), CD3 (FITC), and CD4 (PE). Following fixation and permeabilization using a commercial buffer (BD Biosciences), intracellular staining was performed with antibodies against IFN-γ (APC) and IL-4 (PE-Cy7). Stained cells were analyzed using an Agilent NovoCyte 3000 flow cytometer, and data were analyzed using NovoExpress software 1.6.2 (Agilent, Santa Clara, CA, USA). The percentages of IFN-γ^+^ (Th1) and IL-4^+^ (Th2) cells within the CD3^+^CD4^+^ T cell population were determined.

### 4.11. Statistical Analysis

All data are presented as mean ± standard error of the mean (SEM). Statistical analyses were performed using GraphPad Prism 9.0 software. Comparisons among multiple groups were conducted using one-way analysis of variance (ANOVA) followed by an appropriate post hoc test. Comparisons between two groups were made using Student’s *t*-test. A *p*-value of *^/#^ *p* < 0.05, **^/##^ *p* < 0.01, ***^/###^ *p* < 0.001, or ****^/####^ *p* < 0.0001 was considered statistically significant.

## 5. Conclusions

In conclusion, this study successfully isolated GRB-H, a highly sulfated galactan from the marine red alga *Gigartina radula*, which was hypothetically regarded as a hybrid carrageenan containing comparable amounts of κ- and λ-type structural units. Through integrated in vitro and in vivo analyses, we demonstrated that topical GRB-H effectively alleviates local AD symptoms and mitigates systemic immune dysregulation by modulating serum IgE levels and the Th1/Th2 balance. With its dual anti-inflammatory and immunomodulatory activities and a favorable safety profile compared to a potent glucocorticoid, GRB-H emerges as a promising marine-derived candidate worthy of further development for the topical treatment of atopic dermatitis.

## Figures and Tables

**Table 1:** ^1^H and ^13^C NMR chemical shift assignments of GRB-H in D_2_O at 25 °C (δ in ppm).

Carrageenan	Unit	Chemical Shift (ppm) Relative to DSS as Internal Standard					
		H1/C1	H2/C2	H3/C3	H4/C4	H5/C5	H6/C6
κ	G4S	4.65/104.73	3.60/71.67	4.00/80.41	4.85/76.04	3.82/77.14	3.80/63.55
	DA	5.09/96.92	4.13/71.89	4.53/81.45	4.59/80.72	4.65/79.01	4.05/71.71 4.22/71.73
ι	G4S	4.65/104.73	3.65/75.41	-	4.91/74.24	-	3.80/63.55
	DA2S	5.28/94.29	-	4.53/78.94	-	4.68/77.21	4.01/75.43
μ	G4S	4.68/106.95	3.69/72.84	-	4.92/78.53	3.80/74.33	3.80/63.55
	D6S	5.23/100.39	-	-	-	-	4.14/69.35
ν	G4S	4.68/106.95	3.69/72.84	-	4.92/78.53	3.91/82.30	3.80/63.55
	D2S,6S	5.52/100.39	4.70/80.68	4.00/72.69	4.36/70.01	4.34/82.17	4.28/70.10
λ	G2S	4.84/105.78	-	-	-	-	3.80/63.30
	D2S,6S	5.58/-	-	-	-	-	4.28/71.88

## Data Availability

The data presented in this study are available on request from the corresponding authors.

## Supplementary Materials

The following supporting information can be downloaded at: https://www.mdpi.com/article/10.3390/md24030119/s1. Table S1: Primer sequences; Figure S1: The carrageenan structural units identified in GRB-H.

## References

[1] Ahn C., Huang W. (2017). Clinical Presentation of Atopic Dermatitis. Adv. Exp. Med. Biol..

[2] Tian J., Zhang D., Yang Y., Huang Y., Wang L., Yao X., Lu Q. (2023). Global Epidemiology of Atopic Dermatitis: A Comprehensive Systematic Analysis and Modelling Study. Br. J. Dermatol..

[3] Furue M., Chiba T., Tsuji G., Ulzii D., Kido-Nakahara M., Nakahara T., Kadono T. (2017). Atopic Dermatitis: Immune Deviation, Barrier Dysfunction, IgE Autoreactivity and New Therapies. Allergol. Int..

[4] Çetinarslan T., Kümper L., Fölster-Holst R. (2023). The Immunological and Structural Epidermal Barrier Dysfunction and Skin Microbiome in Atopic Dermatitis—An Update. Front. Mol. Biosci..

[5] Wang W., Shao L., Jiang X., Chu Q., Xiao J., Wu S. (2025). Therapy and Mechanism of Natural Polysaccharides for Atopic Dermatitis Treatment. Food Sci. Hum. Wellness.

[6] Howell M.D., Kim B.E., Gao P., Grant A.V., Boguniewicz M., Debenedetto A., Schneider L., Beck L.A., Barnes K.C., Leung D.Y. (2007). Cytokine Modulation of Atopic Dermatitis Filaggrin Skin Expression. J. Allergy Clin. Immunol..

[7] Hammad H., Lambrecht B.N. (2015). Barrier Epithelial Cells and the Control of Type 2 Immunity. Immunity.

[8] Mancuso J.B., Lee S.S., Paller A.S., Ohya Y., Eichenfield L.F. (2021). Management of Severe Atopic Dermatitis in Pediatric Patients. J. Allergy Clin. Immunol. Pract..

[9] Geng R.S.Q., Sibbald R.G. (2024). Atopic Dermatitis: Clinical Aspects and Treatments. Adv. Skin. Wound Care.

[10] Puar N., Chovatiya R., Paller A.S. (2021). New Treatments in Atopic Dermatitis. Ann. Allergy Asthma Immunol..

[11] Fernando I.P.S., Kim K.-N., Kim D., Jeon Y.-J. (2018). Algal Polysaccharides: Potential Bioactive Substances for Cosmeceutical Applications. Crit. Rev. Biotechnol..

[12] Hu J.H., Yao W.Z., Chang S.Y., You L., Zhao M., Cheung P.C.-K., Hileuskaya K. (2022). Structural Characterization and Anti-Photoaging Activity of a Polysaccharide from *Sargassum fusiforme*. Food Res. Int..

[13] Gescher K., Deters A.M. (2011). *Typha latifolia* L. Fruit Polysaccharides Induce the Differentiation and Stimulate the Proliferation of Human Keratinocytes in Vitro. J. Ethnopharmacol..

[14] Fernando I.P.S., Dias M., Madusanka D.M.D., Han E.J., Kim M.J., Heo S.-J., Lee K., Cheong S.H., Ahn G. (2021). Low Molecular Weight Fucoidan Fraction Ameliorates Inflammation and Deterioration of Skin Barrier in Fine-Dust Stimulated Keratinocytes. Int. J. Biol. Macromol..

[15] Ngatu N.R., Motoyama K., Nishimura Y., Okajima M.K., Hirota R., Higashi T., Lee S., Arima H., Ikeda M., Nojima S. (2017). Anti-Allergic and Profilaggrin (ProFLG)-mRNA Expression Modulatory Effects of Sacran. Int. J. Biol. Macromol..

[16] Archer N.K., Jo J.H., Lee S.K., Kim D., Smith B., Ortines R.V., Wang Y., Marchitto M.C., Ravipati A., Cai S.S. (2019). Injury, Dysbiosis, and Filaggrin Deficiency Drive Skin Inflammation through Keratinocyte IL-1α Release. J. Allergy Clin. Immunol..

[17] Chen B.R., Hsu K.T., Li T.L., Chan Y.-L., Wu C.-J. (2021). Topical Application of Fucoidan Derived from *Cladosiphon okamuranus* Alleviates Atopic Dermatitis Symptoms through Immunomodulation. Int. Immunopharmacol..

[18] Lee T.-K., Kim D.W., Ahn J.H., Lee C.-H., Lee J.-C., Lim S.S., Kang I.-J., Hong S., Choi S.Y., Won M.-H. (2022). Protective Effects of Topical Administration of Laminarin in Oxazolone-Induced Atopic Dermatitis-like Skin Lesions. Mar. Drugs.

[19] Motoyama K., Tanida Y., Sakai A., Higashi T., Kaneko S., Arima H. (2018). Anti-Allergic Effects of Novel Sulfated Polysaccharide Sacran on Mouse Model of 2,4-Dinitro-1-fluorobenzene-Induced Atopic Dermatitis. Int. J. Biol. Macromol..

[20] Zhang D., Wei Y., Zhu X., Zong L., Cui M., Li D., Zhang C. (2025). Study on the Intervention Mechanism of *Ganoderma lucidum* Polysaccharides in Mice with Atopic Dermatitis. Food Res. Int..

[21] He X., Yamauchi A., Nakano T., Yamaguchi T., Ochiai Y. (2019). The Composition and Anti-Inflammatory Effect of Polysaccharides from the Red Alga *Chondrus verrucosus*. Fish. Sci..

[22] Chaves L.S., Nicolau L.A.D., Silva R.O., Barros F.C.N., Freitas A.L.P., Aragão K.S., Ribeiro R.d.A., Souza M.H.L.P., Barbosa A.L.d.R., Medeiros J.-V.R. (2013). Antiinflammatory and Antinociceptive Effects in Mice of a Sulfated Polysaccharide Fraction Extracted from the Marine Red Algae *Gracilaria caudata*. Immunopharmacol. Immunotoxicol..

[23] Costa L.E.C., Brito T.V., Damasceno R.O.S., Sousa W.M., Barros F.C.N., Sombra V.G., Júnior J.S.C., Magalhães D.A., Souza M.H., Medeiros J.-V.R. (2020). Chemical Structure, Anti-Inflammatory and Antinociceptive Activities of a Sulfated Polysaccharide from *Gracilaria intermedia* Algae. Int. J. Biol. Macromol..

[24] Pei Y., Yang S., Xiao Z., Zhou C., Hong P., Qian Z.-J. (2021). Structural Characterization of Sulfated Polysaccharide Isolated from Red Algae (*Gelidium crinale*) and Antioxidant and Anti-Inflammatory Effects in Macrophage Cells. Front. Bioeng. Biotechnol..

[25] Pereira L., van de Velde F. (2011). Portuguese carrageenophytes: Carrageenan composition and geographic distribution of eight species (*Gigartinales*, *Rhodophyta*). Carbohydr. Polym..

[26] van de Velde F., Pereira L., Rollema H.S. (2004). The Revised NMR Chemical Shift Data of Carrageenans. Carbohydr. Res..

[27] Kim J.-Y., Hong J.-E., Woo S.-H., Rhee K.-J., Kim Y.S., Lee Y.-H. (2024). Effect of Pulsed Electromagnetic Field Stimulation on Splenomegaly and Immunoglobulin E Levels in 2,4-Dinitrochlorobenzene-Induced Atopic Dermatitis Mouse Model. Appl. Sci..

[28] Tsiakas S., Angelousi A., Benetou V., Orfanos P., Xagas E., Boletis J., Marinaki S. (2024). Hypothalamic–Pituitary–Adrenal Axis Activity and Metabolic Disorders in Kidney Transplant Recipients on Long-Term Glucocorticoid Therapy. J. Clin. Med..

[29] Hale J., Gerhäuser J., Gaukel V., Wefers D. (2024). Commercially available carrageenans show broad variation in their structure, composition, and functionality. Eur. Food Res. Technol..

[30] Perez Recalde M., Canelón D.J., Compagnone R.S., Matulewicz M.C., Cerezo A.S., Ciancia M. (2016). Carrageenan and agaran structures from the red seaweed *Gymnogongrus tenuis*. Carbohydr. Polym..

[31] Wang P., Zhao X., Lv Y., Li M., Liu X., Li G., Yu G. (2012). Structural and compositional characteristics of hybrid carrageenans from red algae *Chondracanthus chamissoi*. Carbohydr. Polym..

[32] Hughes M.H., Prado H.J., Rodríguez M.C., Michetti K., Leonardi P.I., Matulewicz M.C. (2018). Carrageenans from *Sarcothalia crispata* and *Gigartina skottsbergii*: Structural Analysis and Interpolyelectrolyte Complex Formation for Drug Controlled Release. Mar. Biotechnol..

[33] Almutairi F.M., Adams G.G., Kök M.S., Lawson C.J., Gahler R., Wood S., Foster T.J., Rowe A.J., Harding S.E. (2013). An analytical ultracentrifugation based study on the conformation of lambda carrageenan in aqueous solution. Carbohydr. Polym..

[34] Moreira R., Chenlo F., Torres M.D. (2016). Gelling characteristics and rheology of kappa/iota-hybrid carrageenans extracted from *Mastocarpus stellatus* dried at different temperatures. J. Appl. Phycol..

[35] Hou C., Chen L., Yang L., Ji X. (2020). An Insight into Anti-inflammatory Effects of Natural Polysaccharides. Int. J. Biol. Macromol..

[36] Chen X., Ni L., Fu X., Wang L., Duan D., Huang L., Xu J., Gao X. (2025). Sulfated Undaria pinnatifida Polysaccharides Inhibit Kidney Stone Formation through Crystalline Modulation and Relieving Cellular Oxidative Damage and Inflammation. Biomater. Sci..

[37] Ye J., Zheng L., Pan W., Huang Y., Zhang N., Yang D., Yang Y., Zheng B., Zhang X., Xiao M. (2024). Sulfated Polysaccharide from *Apostichopus japonicus* Viscera Exhibits Anti-inflammatory Properties in Vitro and in Vivo. Int. J. Biol. Macromol..

[38] Chen X., Ni L., Fu X., Wang L., Duan D., Huang L., Xu J., Gao X. (2021). Molecular Mechanism of Anti-Inflammatory Activities of a Novel Sulfated Galactofucan from *Saccharina japonica*. Mar. Drugs.

[39] Huang H., Wang Q., Ning Z., Ma Y., Huang Y., Wu Y., Yang Y., Xiao M., Ye J. (2024). Preparation, Antibacterial Activity, and Structure–Activity Relationship of Low Molecular Weight κ-Carrageenan. Int. J. Biol. Macromol..

[40] Xie X.T., Zhang X., Liu Y., Chen X.-Q., Cheong K.-L. (2020). Quantification of 3,6-Anhydro-galactose in Red Seaweed Polysaccharides and Their Potential Skin-Whitening Activity. 3 Biotech.

[41] López-Abente J., Bernaldo-de-Quirós E., Camino M., Gil N., Panadero E., Campos-Domínguez M., Seoane-Reula E., Gil-Jaurena J.M., Pion M., Correa-Rocha R. (2019). Immune Dysregulation and Th2 Polarization Are Associated with Atopic Dermatitis in Heart-Transplant Children: A Delicate Balance between Risk of Rejection or Atopic Symptoms. Am. J. Transplant..

[42] Gangane P., Sharma V., Selokar M., Vidhate D., Pawar K., Mahajan N. (2024). A review of anti-inflammatory phytoconstituents used in herbal cosmeceuticals for the treatment of atopic dermatitis. Curr. Drug Deliv..

[43] De Simoni E., Candelora M., Belleggia S., Rizzetto G., Molinelli E., Capodaglio I., Ferretti G., Bacchetti T., Offidani A., Simonetti O. (2024). Role of antioxidants supplementation in the treatment of atopic dermatitis: A critical narrative review. Front. Nutr..

[44] Ji H., Li X.K. (2016). Oxidative stress in atopic dermatitis. Oxid. Med. Cell Longev..

[45] Kadri S., Tuvikene R. (2020). Anticoagulant and antioxidant activity of lambda- and theta-carrageenans of different molecular weights. Bioact. Carbohydr. Diet. Fibre.

[46] Sokolova E.V., Barabanova A.O., Bogdanovich R.N., Khomenko V., Solov’eVa T., Yermak I. (2011). In vitro antioxidant properties of red algal polysaccharides. Biomed. Prev. Nutr..

[47] Pala V., Rosset F., Mastorino L., Sciamarrelli N., Boskovic S., Borriello S., Bongiovanni E., Crespi O., Ribero S., Quaglino P. (2025). The Central Role of Th2 Immune Response in Inflammatory Dermatoses: From Pathogenesis to Targeted Therapies. Int. J. Mol. Sci..

[48] Brandt E.B., Sivaprasad U. (2011). Th2 Cytokines and Atopic Dermatitis. J. Clin. Cell. Immunol..

[49] Kondo S., Yazawa H., Jimbow K. (2001). Reduction of Serum Interleukin-5 Levels Reflect Clinical Improvement in Patients with Atopic Dermatitis. J. Dermatol..

[50] Cheung P.F.-Y., Wong C.-K., Ho A.W.-Y., Hu S., Chen D.-P., Lam C.W.-K. (2010). Activation of Human Eosinophils and Epidermal Keratinocytes by Th2 Cytokine IL-31: Implication for the Immunopathogenesis of Atopic Dermatitis. Int. Immunol..

[51] Lim C., Kim N., Kim J., Park H., Lee H., Hong J., Kwon D.O., Choi K., Yoon D. (2025). MMPP Attenuates the Inflammatory Response by Suppressing ROS and Proinflammatory Cytokine and Chemokine Production in TNF-α/IFN-γ-Stimulated Human Keratinocytes. J. Microbiol. Biotechnol..

[52] Suárez-Fariñas M., Dhingra N., Gittler J., Shemer A., Cardinale I., de Guzman Strong C., Krueger J.G., Guttman-Yassky E. (2013). Intrinsic Atopic Dermatitis Shows Similar TH2 and Higher TH17 Immune Activation Compared with Extrinsic Atopic Dermatitis. J. Allergy Clin. Immunol..

[53] Koga C., Kabashima K., Shiraishi N., Kobayashi M., Tokura Y. (2008). Possible Pathogenic Role of Th17 Cells for Atopic Dermatitis. J. Investig. Dermatol..

[54] Ma X., Deng G., Tian N., Wang H., Zhao H., Kuai L., Luo Y., Gao C., Ding X., Li B. (2024). Calycosin Enhances Treg Differentiation for Alleviating Skin Inflammation in Atopic Dermatitis. J. Ethnopharmacol..

[55] Qiu W.-L., Chao C.-H., Hsu Y.-C., Lu M.-K. (2024). Anti-inflammatory Potential of Low-Molecular-Weight and High-Sulfation-Degree Sulfated Polysaccharides Extracted from *Antrodia cinnamomea*. Int. J. Biol. Macromol..

