# Supplementary material for: A λ-Carrageenan-Enriched Sulfated Galactan from Gigartina radula Attenuates Atopic Dermatitis via Coordinated Anti-Inflammatory and Immunomodulatory Mechanisms
Source: Mar Drugs. 2026 Mar 22;24(3):119. doi: 10.3390/md24030119 (PMC13028040; doi:10.3390/md24030119)
Supplement: Supplementary file 1 [file marinedrugs-24-00119-s001.zip › marinedrugs-4212604-supplementary.pdf]

Supplementary material

A λ-Carrageenan-Enriched Sulfated Galactan from *Gigartina radula* Attenuates Atopic Dermatitis via Coordinated Anti-inflammatory and Immunomodulatory Mechanisms

Kexin Du[1], Shuo Liang[1], Zijing Wu[1,3], Yujing Wang[1], Pengcheng Gao[1], Wei Han[1,3], Youjing Lv[1,*], Guangli Yu[1,2], Guoyun Li[1,2,*]

[1] Key Laboratory of Marine Drugs, Ministry of Education, School of Medicine and Pharmacy, Shandong Key Laboratory of Glycoscience and Glycotherapeutics, Ocean University of China, Qingdao 266003, China

[2] Laboratory for Marine Drugs and Bioproducts, Qingdao Marine Science and Technology Center, Qingdao 266237, China.

[3] Qingdao Key Laboratory of Respiratory Comorbidity Remodeling and Precision Prevention, Qingdao Municipal Hospital, Qingdao 260071, China

* Correspondence: liguoyun@ouc.edu.cn (G.L.); 2015315@ouc.edu.cn (Y.L.)

Table S1. Primer sequences

Gene name	**Usage**	**Sequences**
GAPDH	F primer	5′-GGTGAAGGTCGGTGTGAACGGATT-3′
	R primer	5′-AATGCCAAAGTTGTCATGGATGACC-3′
TNF-α	F primer	5′-AACATCCAACCTTCCCAAACG-3′
	R primer	5′-CTCTTAACCCCCGAACTCCCAG-3′
IFN-γ	F primer	5′-GAAAGCCTAGAAAGTCTGAATAACT-3′
	R primer	5′-ATCAGCAGCGACTCCTTTTCCGCTT-3′
IL-4	F primer	5′-TCGGCATTTTGAACGAGGTC-3′
	R primer	5′-GAAAAGCCCGAAAGAGTATC-3′
IL-5	F primer	5′-ATGGAGATTCCCATGAGCAC-3′
	R primer	5′-GTCTCTCCTCGCCACACTTC-3′
IL-6	F primer	5′-TGGAGTCACAGAAGGAGTGGCTAAG-3′
	R primer	5′-TCTGACCACAGTGAGGAATGTCCAC-3′
IL-31	F primer	5′-TCGGTCATCATAGCACATCTGGAG-3′
	R primer	5′-GCACAGTCCCTTTGGAGTTAAGTC-3′

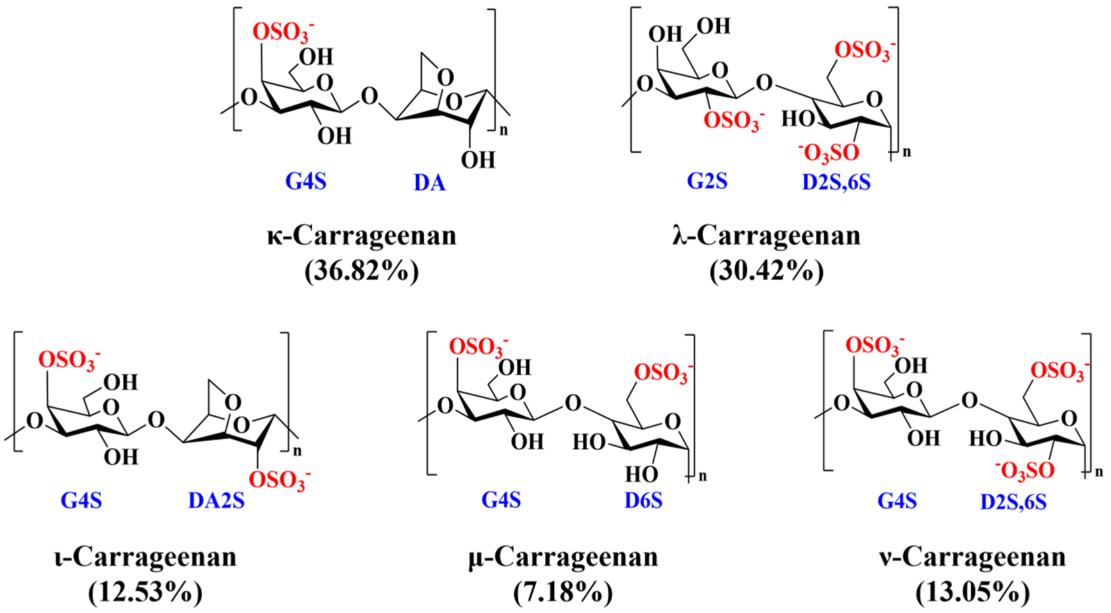

Figure S1. The carrageenan structural units identified in GRB-H
